# Barriers to cognitive screening in acute stroke units

**DOI:** 10.1038/s41598-021-98853-5

**Published:** 2021-10-04

**Authors:** Tamar Abzhandadze, Dongni Buvarp, Åsa Lundgren-Nilsson, Katharina S. Sunnerhagen

**Affiliations:** 1 Institute of Neuroscience and Physiology, The Sahlgrenska Academy, University of Gothenburg, Per Dubbsgatan 14, fl. 3, 413 45 Gothenburg, Sweden; 2 Department of Occupational Therapy and Physiotherapy, Sahlgrenska University Hospital, Bruna Stråket 11 B. 413 46, Gothenburg, Sweden

**Keywords:** Stroke, Neurology, Health care, Stroke

## Abstract

Cognitive impairment is common after stroke. However, not all patients with stroke undergo cognitive screening, despite recommendations. The aim of this retrospective, explorative study was to examine the barriers to cognitive screening in acute stroke units. Data were retrieved from two Swedish Stroke registries. The outcome variable was cognitive screening during the stay at acute stroke units. Forty-three candidate explanatory variables were considered for analysis, encompassing sociodemographic factors and stroke-related outcomes during the stay at acute stroke units. The Least Absolute Shrinkage and Selection Operator and decision-tree methods were used. Of the 1120 patients (56% male, mean age: 72 years, 50% with mild stroke), 44% did not undergo cognitive screening. Walking 10 m post-stroke was the most important attribute for decisions regarding cognitive screening. The classification accuracy, sensitivity, and specificity of the model were 70% (95% CI 63–75%), 71% (63–78%), and 67% (55–77%), respectively. Patient-related parameters that influenced cognitive screening with a valid and reliable screening instrument in acute stroke units included new stroke during the hospitalisation, aphasia at admission, mobility problems, impaired verbal output skills, and planned discharge to another care facility. The barriers to cognitive screening were both patient- and organisation-related, suggesting the need for patient-tailored cognitive screening tools as well as the implementation and systematic adherence to guidelines.

## Introduction

Cognitive impairments are common sequelae after stroke^1,2^ and are also associated with dependency in everyday life^3^, problems returning to work^4^, and higher incidence of depression^5^. Cognitive impairment early after stroke is a consequence of interactions between size and localisation of the lesion, pre-morbid cognitive status, and sociodemographic factors^6^. In some cases, cognitive deficits are obvious in clinical practice; however, patients with mild or subtle cognitive problems could be missed and discharged without appropriate assessment.

Patients with clinically evident stroke should be considered to have a risk for developing cognitive impairment and offered cognitive assessment prior to discharge from acute stroke units (ASUs)^7^. Comprehensive cognitive assessments in ASUs can be challenging for both patients and healthcare professionals. For patients, cognitive assessments can be stressful. For healthcare professionals, it can be difficult to perform time-consuming comprehensive cognitive assessments during the acute phase of stroke, as the patients need to undergo numerous medical examinations. Therefore, cognitive screening with short, validated screening instruments has been recommended for identifying patients with cognitive impairments and at need for further services and rehabilitation^7,8^.

One of the recommended instruments for cognitive screening is the Montreal Cognitive Assessment (MoCA)^7^. The MoCA is a brief instrument with good sensitivity and specificity for detecting mild cognitive impairment in patients with stroke^7^. However, not all patients undergo even this short cognitive screening. Patients are more likely to not undergo cognitive screening if they are older, have severe stroke, aphasia, impaired function in the dominant upper limb, dementia, and pre-morbid dependency in activities of daily living (ADL)^9–12^. Some studies reporting these findings included relatively small sample sizes of patients with acute and subacute stroke, different sets of available variables, and various statistical analyses. In some studies, data collection was conducted with pre-specified inclusion and exclusion criteria that can lack representativeness in clinical practice.

Clinical practice in ASUs depends on various factors, including local guidelines and availability of rehabilitation services. Therefore, barriers to cognitive screening can vary. By using the register data from ASUs, we aimed to study the barriers to cognitive screening early after stroke and to establish a better understanding of the decision-making process regarding cognitive screening at ASUs. This knowledge may elucidate why cognitive screening is not being conducted despite recommendations.

## Methods

### Study design and participants

This was a retrospective, explorative study, part of the Physical Activity Pre-Stroke In GOThenburg project^13^. Data from two Swedish stroke registries were used: Väststroke and Riksstroke^13^. Väststroke is a local quality registry for stroke at the Sahlgrenska University Hospital (SU), Gothenburg. The SU provides emergency and basic care for the Gothenburg region, with approximately 700,000 inhabitants, and specialised care for West Sweden, with approximately 1.7 million inhabitants. In Väststroke, the data from three admission sites (hospitals) were included; Reperfusion treatment is provided at one site, according to the regional agreement. Riksstroke is the national quality registry for stroke care. The statistician at Riksstroke linked the data from Väststroke to Riksstroke using the patients’ identification numbers^14^. The major reason for data-linkage was the type of information contained in these registries. Väststroke contains data on patient outcomes early after stroke, including cognition outcomes, and Riksstroke contains other information, including the pre-hospital status and medical treatment. The data were retrieved from 1 November 2014 to 31 August 2018. The discharge pathways from ASUs are similar to each other. When the patients’ medical conditions are stabilised, they can be discharged to their homes with or without home help, residential or long-term national health service (NHS) homes, rehabilitation units (patients can also stay in ASUs for short-term rehabilitation), other hospitals, or other departments in the same hospital. However, most of the patients are usually discharged to their homes and residential or long-term NHS homes.

We included patients with first-ever ischaemic or haemorrhagic stroke, an age > 18 years, and data on cognitive screening (yes/no). Patients were excluded if they died during hospitalisation or if they had incomplete data pertaining to the explanatory variables.

### Ethics

The data file that was used in the study was anonymised, and individual patients could not be identified. The study obtained ethical approval from *the Regional Ethical Review Board in Gothenburg *(346-16, amendment: T807-18) and the research was performed in accordance with all guidelines and regulations. Regarding informed consent, according to the Swedish Data Protection Authority, the handling of data generated within the framework of quality registries is exempt from the general rule requiring written informed consent from patients. Furthermore, the Personal Data Act (Swedish law #1998:204, issued 29 April 1998) allows data from medical charts to be collected for clinical purposes and quality control without written informed consent. Following the Declaration of Helsinki was not relevant to this project, which was based on data that were generated within quality registries.

### Data collection and registration

The data in Väststroke were collected and registered by the multidisciplinary healthcare staff working in three sites of the SU. Medical data were recorded by physicians and nurses. Activity and functional status were assessed and registered by occupational therapists, physiotherapists, and speech therapists^15^. Occupational therapists performed screening of global cognition with the MoCA, the most used and recommended standardised screening tool in ASUs. The Research nurses reported the data for Riksstroke. Patient charts were used as a source of information. The neurological status was assessed by physicians or nurses, and the results of the neurological status at admission to the hospital were registered.

### Variables

The *outcome variabl*e was cognitive screening during the stay at acute stroke units. The variable had two answer alternatives: No, cognition was not screened (coded as 1) and Yes, cognition was screened (coded as 0). Cognition was screened with the MoCA^16,17^—a valid and reliable instrument for the screening of cognition in patients with mild to moderate stroke. The scores range between 0 and 30 points, and ≥ 26 points indicate normal cognition^16,17^. The MoCA was a major valid and reliable instrument used for the screening of global cognition at ASUs during this study period.

#### Explanatory variables

Forty-three variables were identified as relevant based on previous research^9–12^ as well as clinical experience and were included in the analyses. Stroke type was classified according to the International Classification of Diseases criteria; no traumatic intracerebral haemorrhage (I61) and cerebral infarction (I63) were included. The neurological status at admission was assessed using the National Institutes of Health Stroke Scale (NIHSS)^18^. The level of consciousness on arrival at hospital was assessed using the Reaction Level Scale (RLS)^19^. Patients' verbal output skills were screened during the stay at ASUs on a 4-grade scale, where 0 is normal and 4 signifies that the patient cannot collaborate during the assessment (because of severe dysarthria or an inability to communicate in Swedish). Information on sex, age, accommodation and ADL prior to the stroke, comorbidities, length of hospital stay, and reperfusion treatment were also included in the analyses. Detailed definitions and coding of the variables are presented in Supplemental Table I.

### Statistics

Prior to data modelling, explanatory variables were checked for the assumption of a minimum of 10 observations per outcome class, and the ordinal variables were dichotomised if the assumption was not satisfied (Supplemental Table I). The patients who had incomplete data regarding the explanatory variables were excluded from the analyses. This step was necessary to obtain comparable models.

To identify the variables that could explain why cognitive screening was not conducted, the Least Absolute Shrinkage and Selection Operator (LASSO) method was used (the first aim)^20–22^. The major advantage of LASSO is to handle large set of the variables with possible multicollinearity by introducing penalty terms. With the penalty terms, LASSO shrinks the coefficients of correlated variables. In addition, by using LASSO we wanted to create a sparse model with few explanatory variables to render the interpretation of the model easier.

In LASSO, the regularisation parameter is lambda with a positive value. As lambda increases, the regression coefficients of the variables are shrinking to 0, and non-important variables are eliminated from the model. The remaining variables are regarded as important.

In this study, the adaptive LASSO method was applied to avoid standardisation of the variables^22^. Prior to analyses, the data were divided into a training set (80%) and a test set (20%); this step was important for studying the stability of the model. The process of model building was as follows. First, all explanatory variables were entered in the analysis without standardisation and penalty parameters (alpha = 1, lambda = 0). This LASSO model provided the ordinary least squares (OLS, [regression coefficient]) coefficients^22^. Second, the intercept from the OLS regression coefficients was extracted. Finally, we built a 10-fold cross-validated adaptive LASSO model by introducing a penalty parameter, one divided by the absolute value of the OLS regression coefficients. The model presented in the study was within one standard error (1 SE) of the lambda value.

To understand why cognitive screening was not performed in different sites (second aim), a decision tree algorithm was used^23^. All explanatory variables were included in the analyses. The data were divided into a training set (80%) and a test set (20%). Potential training biases were avoided by shuffling the data rows. The model-building process was as follows: a large, over-fitted decision tree was created based on the probability of randomly selected individuals being wrongly classified (known as the Gini index). The minimum number of observations for a split to be attempted was set at 5. The complexity parameter was set at 0.0001. The classification accuracy of the models from the training and testing datasets was obtained. *Tree pruning*: 10-fold cross-validation was performed to determine the optimal parameters for pruning. The primary parameter for selecting the model was the minimum value of the cross-validation error (min-xerror). Once the min-xerror was identified, the decision tree was built on the training set and tested thereafter on the test set. The models are presented with classification accuracy, sensitivity, specificity, and 95% confidence intervals (95% CI).

The univariable binary logistic regression analyses were performed to elucidate the importance of the explanatory variables. From the analyses, we have obtained odds ratios, 95% confidence intervals, p-values and the Area Under the Curve (AUC, values ≤ 0.5 indicate a poor fit^24^) values for each explanatory variable. The outcome variable was defined as not having cognitive screening (coded as 1).

The data were analysed using SPSS (IBM Corp. Released 2019. IBM SPSS Statistics for Windows, Version 26.0. Armonk, NY). GraphPad prism (Version 9, GraphPad Software, Inc., http://www.graphpad.com) was used for figures. The licences for SPSS and GraphPad prism were provided by the University of Gothenburg. R software was also used (R Core Team, version 4.0.2; R Foundation for Statistical Computing, Vienna, Austria). R can be downloaded free of charge at https://cran.r-project.org/bin/windows/base/. The significance level for two-tailed tests was set at an alpha level of 5%.

## Results

**Figure 1:**
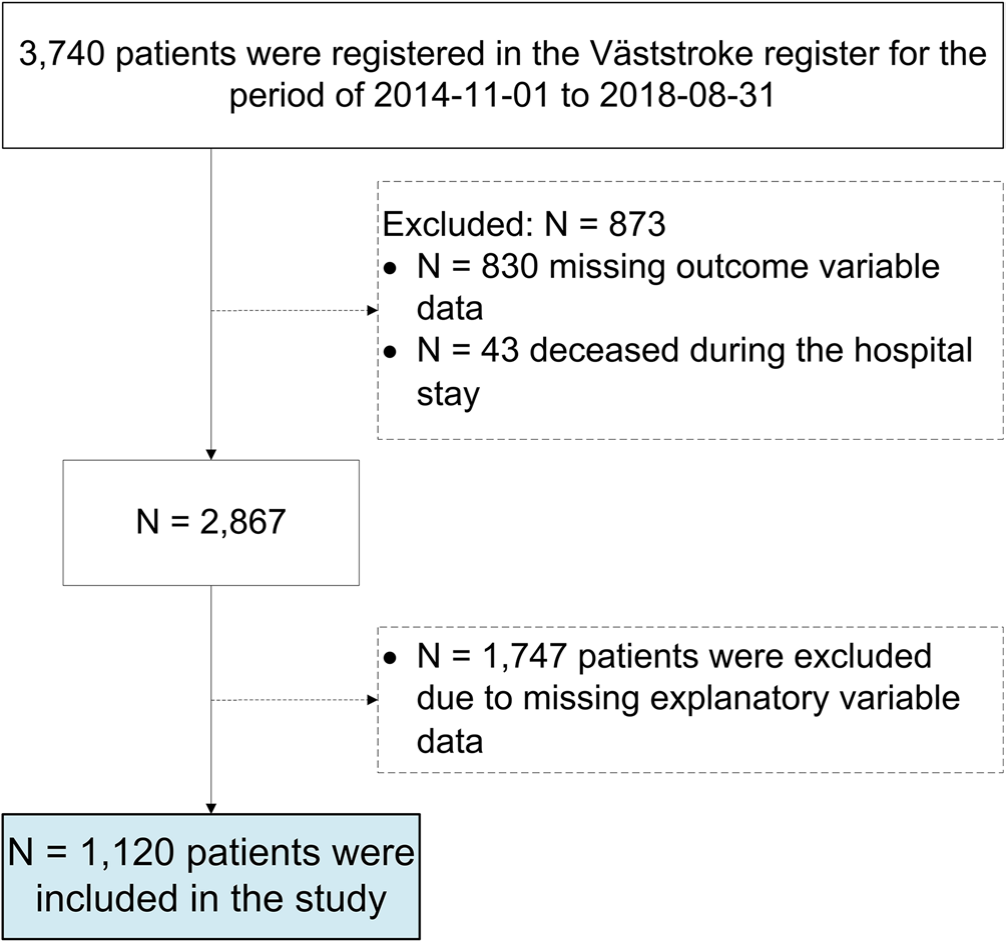
Flowchart of the study participants.

In total, 1120 patients met the inclusion criteria from the dataset comprising 3740 patients. The major reason for exclusion was missing data on any of the explanatory variables used for model building (Fig. 1). Among the excluded patients (N = 2620), compared with the included ones (N = 1120), there were more males (p = 0.001), they had higher mean ages (p < 0.001), and they had a higher median NIHSS score (p < 0.001).

Of the 1120 patients included in the study, 493 (44%) were female, the mean age was 72 years (range, 19–100 years), 50% had a mild stroke (NIHSS ≤ 3), and the median length of hospital stay was 8 days (range, 1–100 days). Patients without cognitive screening (N = 488), compared with the patients with cognitive screening (N = 632), were older (p = 0.002), had a greater need for help before stroke (p < 0.001), and had a 1-point higher median NIHSS score (p < 0.001) (Table 1). Patients admitted to the stroke unit where reperfusion treatment was available had a lower mean age (p < 0.001) and more severe stroke (p < 0.001) than those admitted to other stroke units (Supplemental Table II).

**Table 1:** Characteristics of the study participants (N = 1120).

	All N = 1120	Cognitive screening		p-value
		Yes, N = 632	No, N = 488	
Male, n (%)	626 (56)	367 (58)	260 (41)	0.11^†^
Age, years, mean ± s.d.	72 ± 14	71 ± 13	73 ± 14	**0.002**
Median (min–max)	74 (19–100)	73 (20–99)	75 (19–100)	
TIA^‡^ prior to stroke, yes, n (%)	72 (6)	35 (5)	37 (7)	0.17^†^
Diabetes, yes, n (%)	192 (17)	104 (16)	88 (18)	0.49^†^
Atrial fibrillation, yes, n (%)	227 (20)	125 (20)	102 (21)	0.64^†^
Lived alone prior to stroke, n (%)	528 (47)	283 (45)	245 (50)	0.07^†^
Needed help prior to stroke, n (%)	120 (11)	41 (6)	79 (16)	**< 0.001**^†^
ADL-independent prior to stroke, n (%)	1051 (94)	613 (97)	438 (89)	**< 0.001**^†^
**Stroke diagnosis n (%)**				0.58^†^
I 61 Cerebral haemorrhage	31 (3)	19 (3)	12 (2)	
I 63 Cerebral infarctions	1089 (97)	613 (97)	476 (98)	
**Admission site, n (%)**				**< 0.001**^†^
Site A	230 (20)	167 (26)	63 (13)	
Site B—with reperfusion treatment	513 (46)	256 (41)	257 (53)	
Site C	377 (34)	209 (33)	168 (34)	
Reperfusion treatment, yes n (%)	159 (14)	74 (12)	85 (17)	**0.007**^†^
Had recurrent stroke, n (%)	50 (5)	16 (2)	34 (7)	**< 0.001**^†^
**Level of consciousness at admission, n (%)**				**0.05**
Fully awake (RLS ^§^ 1)	1081 (97)	616 (97)	465 (95)	
Drowsy or unconscious (RLS 2–8)	39 (3)	16 (3)	23 (5)	
NIHSS^¶^, median (range)	1 (0–28)	1 (0–24)	2 (0–28)	**< 0.001**
**Verbal output skills, n (%)**				**< 0.001**
Normal	430 (38)	266 (42)	164 (34)	
Can be understood	260 (23)	164 (26)	96 (20)	
Needs questions and help for communication	97 (9)	31 (5)	66 (13)	
Can partly communicate, but unsure	47 (4)	3 (0.5)	44 (9)	
Cannot collaborate enough for the conclusion	18 (2)	1 (0.5)	17 (3)	
Cannot communicate in any way	268 (24)	167 (26)	101 (21)	
Cognitive function assessed with the MoCA^††^, median (range)		25 (8–30)		
Length of hospital stay, days, mean ± s.d./median (range)	13 ± 14/8 (1–100)	11 ± 11/7 (2–100)	16 ± 17/9 (1–100)	**0.008**
**Discharge destination, n (%)**				**< 0.001**^†^
Own home with/without community services	882 (79)	558 (88)	324 (66)	
Community facility/other hospitals or units	238 (21)	74 (12)	164 (34)	

p-value: Statistical difference between the patients with (N = 632) and without (N = 488) cognitive screening; the bold text indicates statistically significant results. ^†^Chi-squared test or Mann-Whitney U test. ^‡^*TIA* transient ischemic attack, ^§^*RLS* Reaction Level Scale, ^¶^*NIHSS* National Institutes of Health Stroke Scale, ^††^*MoCA* Montreal Cognitive Assessment.

### Barriers to cognitive screening: explanatory attributes

**Figure 2:**
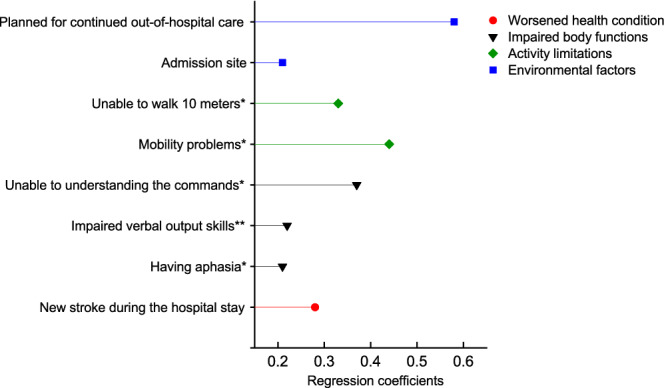
The regression coefficients of the 10-fold cross-validated adaptive least absolute shrinkage and selection operator model. *Indicates post-stroke conditions.

The 10-fold cross-validated adaptive LASSO model yielded a lambda coefficient of 0.5 (within 1 SE) and a degree of freedom of 8. Eight variables (regression coefficients) were found for not receiving cognitive screening: new stroke during the hospital stay (0.28), aphasia/NIHSS sub-item (0.21), inability to understand the commands/NIHSS sub-item (0.37), mobility problems (0.44), impaired verbal output (0.22), inability to walk 10 m post-stroke (0.33), admission site (0.21), and patients planned for continued out-of-hospital care (0.58) (Fig. 2).

### Cognitive screening: decision-making process

**Figure 3:**
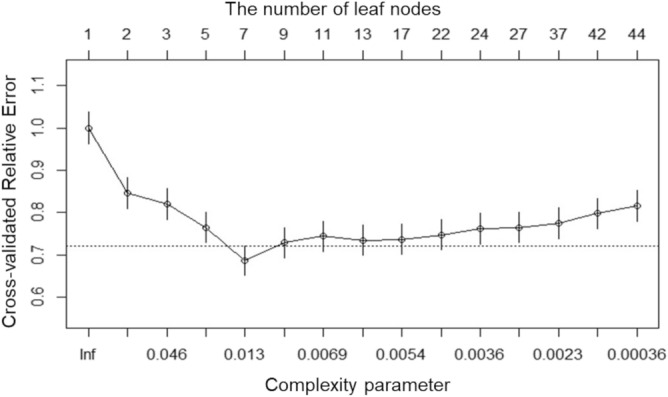
Parameters of the 10-fold cross-validated decision tree model. The primary parameter for selecting the model is the minimum value of the cross-validated relative error (the lowest value is selected in further analyses). R, version 4.0.2, https://www.rstudio.com.

The full, over-fitted decision tree model including all independent variables, had classification accuracies of 78% and 67% for the training and testing datasets, respectively. These results indicate the poor performance of the model. The tenfold cross-validated decision tree model provided a min-xerror value of 0.69 (SD, 0.03) (Fig. 3), yielding a complexity parameter of 0.009, number of leaf nodes of 7, and number of splits of 5.

**Figure 4:**
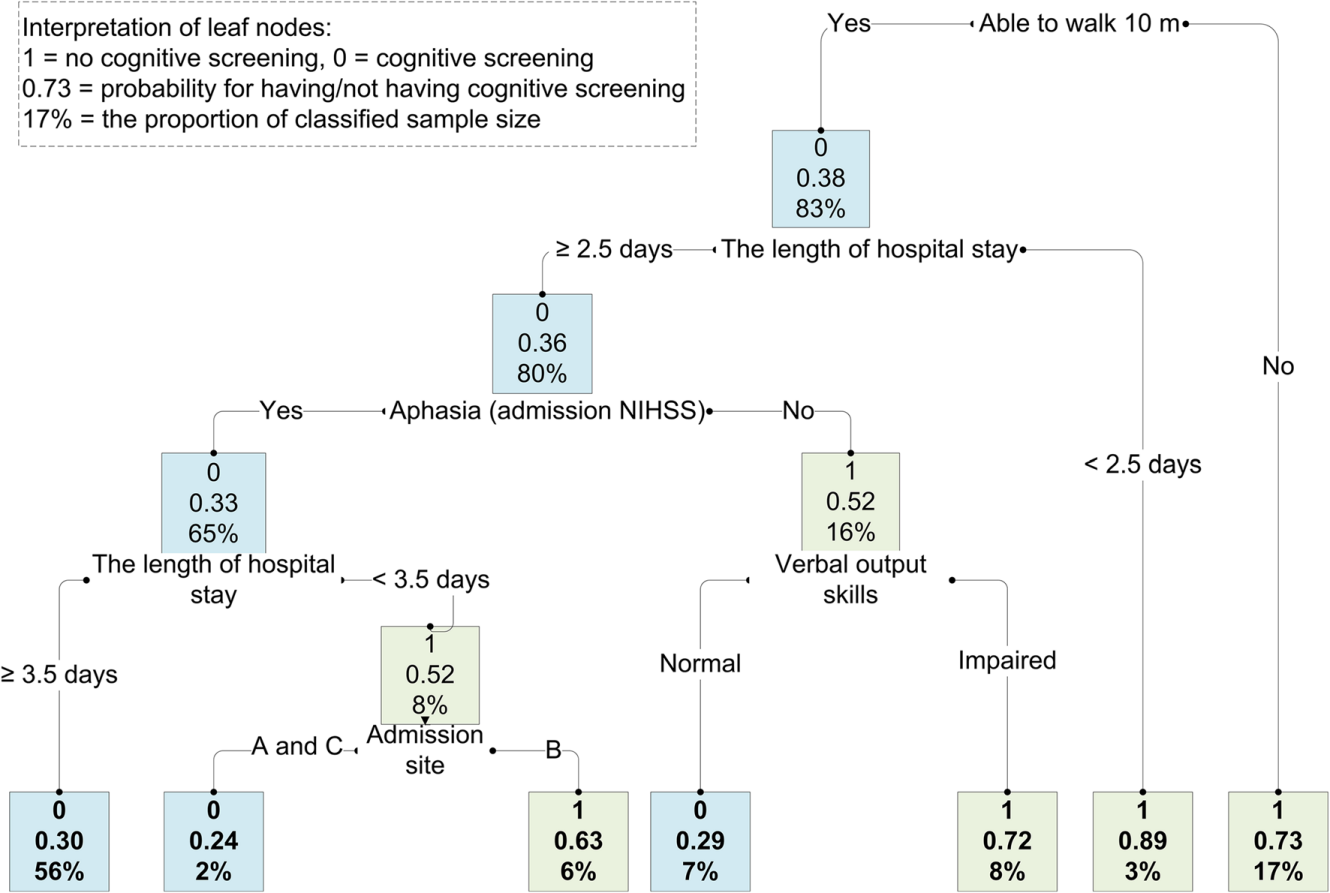
Decision tree with seven leaf nodes representing the decision-making process for cognitive screening. *NIHSS* The National Institutes of Health Stroke Scale, *A and C* admission sites without reperfusion treatment, *B* admission site with reperfusion treatment.

The pruned decision tree model based on the min-xerror showed that the training data had a classification accuracy, sensitivity, and specificity of 71% (95% CI 68–74%), 70% (66–73%), and 74% (68–79%), respectively. The test data provided a classification accuracy, sensitivity, and specificity of 70% (95% CI 63–75%), 71% (63–78%), and 67% (55–77%), respectively. Walking 10 m post-stroke was the most important attribute. Patients who could not walk 10 m had a 73% probability of not having cognitive screening, and 17% of the sample was classified. The patients who could walk 10 m but stayed in the ASU for less than 2.5 days had an 89% probability of not undergoing cognitive assessment, and 3% of the sample was classified (Fig. 4).

### Explaining barriers to cognitive screening

**Figure 5:**
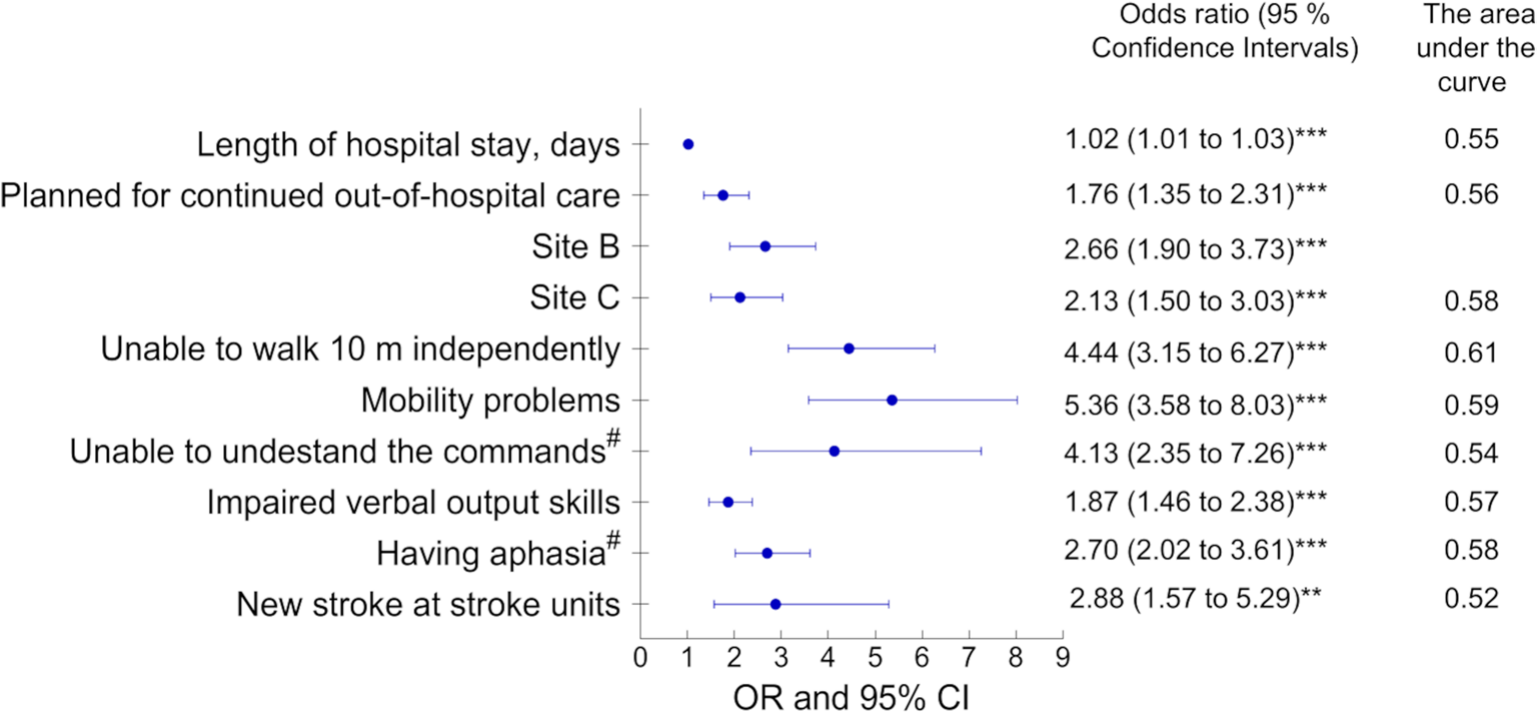
A forest plot showing the results of univariable binary logistic regression analyses explaining the barriers to cognitive screening in 1120 patients with first-ever stroke. Site B provides reperfusion treatment.

The univariable binary logistic regression analyses were performed based on the variables that were selected by the LASSO and decision tree analyses (Fig. 5). All variables were significant, and the odds ratio of not having cognitive screening varied from 1.02 to 5.36 increase per independent variable. AUC values were low, but acceptable.

## Discussion

This clinical practice, data-based study showed that 44% of the patients did not undergo cognitive screening during their stay in ASUs. The proportion of unscreened patients is higher than those reported by other studies, where approximately 17% of the patients did not undergo cognitive screening^10,25^. The divergence between the results of the present and previous studies may be related to the study design. In our study, the registry datasets generated from clinical practice were used. Thus, a more representative picture of clinical practice could be described compared with that in other studies where data collection was conducted with pre-specified inclusion and exclusion criteria^10,25^. Therefore, the results might be representative of other stroke units. Furthermore, the patients in our study had less severe stroke than did the patients in the other studies^10,25^ and were possibly regarded as cognitively intact.

There are different ways to perform cognitive screenings in ASUs. One way is via the standardised screening tools, and another is via observations of activity performance. It is possible that patients who were not screened with MoCA received cognitive screenings during observations of activity performance. Although such observations give ecologically valid pictures of how impaired cognition impacts a patient’s functioning, the results can have reliability problems. Moreover, since one patient can meet different personnel during their stay at ASUs and when discharged from the hospital, it might be hard to track the recovery process without objective measures. Therefore, combining both approaches is important, as they provide different types of information on cognitive function after stroke.

Patients with recurrent stroke at the ASUs did not undergo cognitive screening. It is possible that patients’ neurological status had worsened after a recurrent stroke^26^; therefore, cognitive screening was not prioritised, given the necessity to address other medical issues. Furthermore, impaired verbal output skills negatively affected cognitive screening. Our study results were partly in line with those of other studies^10,25^. The applicability of the MoCA depends on adequate communication skills and good function in the dominant upper limb^27^. This can be a limitation for screening instruments used in stroke units. Therefore, other stroke-specific screening instruments and several short versions of the MoCA have been developed^28^. However, very few of them have been translated into Swedish. Perhaps by having different standardised instruments available, more patients would undergo cognitive screenings.

A walking capacity of 10 m post-stroke was the most important attribute for the decision-making process; patients who could not walk independently were less likely to undergo cognitive screening. It is possible that patients with limited ability to move independently had more severe motor impairments and an increased need for help. Thus, it can be assumed that the focus and priority for assessments and rehabilitation was to manage those impairments^29^. However, there is a risk of overlooking cognitive impairment in this group because movement involves cognition^17^. Furthermore, patients who could walk 10 m post-stroke but had a short length of stay at ASUs had a high probability of not receiving cognitive screening with the MoCA. One explanation is that these patients were considered to have no neurological or cognitive sequelae and were discharged early. It is also possible that there was no time for cognitive screening within a short length of stay.

There was a difference between admission sites regarding the proportion of patients not receiving cognitive screening. Although all sites at the SU have the same guidelines for cognitive screening, it is possible that there are local derivations from these guidelines. Moreover, our results could depend on the workforce, such as a shortage of staff and perhaps the experience of the healthcare professionals responsible for cognitive screening. Another explanation could be patient related^10,27^; the stroke unit with the highest proportion of patients with severe stroke at admission performed fewer cognitive screenings with the MoCA. It is possible that in these patients, sufficient cognitive impairments were observed; thus, screening with the MoCA was not considered necessary. It is also possible that patients with severe stroke had impaired communication skills and motor functions; thus, the MoCA could not be used. We can assume that many of these patients have undergone cognitive assessment during activity performance, another common practice at ASUs.

There are several strengths and limitations of this study. Adaptive LASSO has good classification accuracy. However, LASSO coefficients are regression coefficients, and we cannot extract probabilities or estimate the hierarchal order between the variables. Therefore, the decision tree algorithm was applied, including all explanatory variables^23^. Our training model achieved 71% classification accuracy. The performance of the model did not deteriorate when testing it in the test set (70%), which indicates that the model performance is relatively good. Moreover, to obtain the information on the influence of each explanatory variable on the outcome, univariable binary logistic regression analyses were performed, where each independent variable was a significant predictor; however the odds ratio varied among the variables. Older patients and patients with more severe stroke were excluded; theoretically, these patients could have cognitive impairments because of advanced age as well as severe stroke and may have been underrepresented in this study. Furthermore, the major reason for excluding the patients from the analyses was missing data on any of the explanatory variables. Perhaps, by using fewer explanatory variables, we could retain a larger proportion of the patients with available data. However, variables included in the study were regarded as clinically important by clinicians and registry holders, as these variables were included in the registries based on the clinical practice and were used for monitoring the quality of stroke care in Sweden. Although, the remaining study sample size was large and representative of the Swedish stroke population in terms of stroke severity, for the results to be generalisable, external validation in cohorts from other healthcare systems will be required. The spectrum of patients was wide and included those with communication difficulties. Hospital-based information from ASUs, guided by the Swedish National Guidelines for Stroke Care and Rehabilitation^15^, was used. Finally, the MoCA, a recommended and feasible screening instrument for cognition in an acute stroke setting^7^, was used for cognitive screening in ASUs; however, it is not suitable for all patients.

## Conclusions

The barriers to cognitive screening at ASUs are partly related to the consequences of stroke. If cognitive screening tools could be better tailored to individual function and activity capacity, a higher proportion of patients could undergo cognitive screening with valid and reliable instruments. Other limitations are related to organisational factors, indicating the need for implementation and adherence to guidelines for cognitive screening. The results indicate the need for clear guidelines for cognitive screenings with standardized, valid, and reliable cognitive screening tools in the acute stroke units, because depending on the workload, some assessments can be deprioritized.

## Supplementary Information

Supplementary Tables.

## Data Availability

The datasets generated and analysed during the current study are not publicly available. According to the Swedish regulations shown in https://etikprovning.se/for-forskare/ansvar/, the data cannot be publicly shared because of ethical and legal reasons. The data are available on reasonable request. Researchers can request access to the data by emailing the principal investigator at ks.sunnerhagen@neuro.gu.se.
