# Supplementary material for: Barriers to cognitive screening in acute stroke units
Source: Sci Rep. 2021 Oct 4;11:19621. doi: 10.1038/s41598-021-98853-5 (PMC8490375; doi:10.1038/s41598-021-98853-5)
Supplement: Supplementary file 1 — Supplementary Tables. [file 41598_2021_98853_MOESM1_ESM.docx]

**Supplementary material**

**Title**: Barriers to cognitive screening in acute stroke units

**Authors**
Tamar Abzhandadze^1,2^, Dongni Buvarp^1^, Åsa Lundgren-Nilsson^1^, Katharina S. Sunnerhagen^1^

^1^Institute of Neuroscience and Physiology, the Sahlgrenska Academy, University of Gothenburg, Per Dubbsgatan 14, fl. 3, 413 45 Gothenburg, Sweden

^2^ Department of Occupational Therapy and Physiotherapy, Sahlgrenska University Hospital, Bruna Stråket 11 B*.* 413 46, Gothenburg, Sweden.

**Corresponding author:**

Tamar Abzhandadze,

Institute of Neuroscience and Physiology,

the Sahlgrenska Academy,

University of Gothenburg,

Per Dubbsgatan 14, fl. 3,

413 45 Gothenburg, Sweden

Fax: +46 317 412 659

E-mail: tamar.abzhandadze@gu.se

Supplemental Table I. Description of the explanatory variables used for model building. The variables were retrieved from Väststroke and Riksstroke registries where patients with first ever stroke were included.

| **Explanatory variables** | **Characteristics of the explanatory variables** | **Characteristics of the explanatory variables entered into the modelling analyses** |
| --- | --- | --- |
| Sex | Binary | Binary: 1 = male, 2 = female |
| Age, years | Scale, range 19-100 | Scale: range 19-100 |
| Accommodation before stroke | Ordinal, 3 levels | Nominal: 1 = own home without community services, 2 = own home with help or community facilities. |
| Lives alone, prior to stroke | Binary, yes/no | Binary: 1 = yes, 2 = no |
| Needed help, prior to stroke | Binary, yes/no | Binary: 1 = no, did not need help, 2 = yes, needed help |
| ADL ability, prior to stroke | Binary, yes/no | Binary: 1 = independent prior to stroke, 2 = dependent prior to stroke |
| Had TIA prior to stroke | Binary, yes/no | Binary: 1 = no, 2 = yes |
| Diabetes | Binary, yes/no | Binary: 1 = no, 2 = yes |
| Atrial fibrillation | Binary, yes/no | Binary: 1 = no, 2 = yes |
| **Stroke-related conditions** | | |
| Level of awareness at admission | Ordinal, 3 levels | Binary: 1 = alert (RLS 1), 2 = drowsy or unconscious (RLS 2 - 8) |
| Admission site at SU | Nominal, 3 levels | Nominal: 1 = SU/Mölndal, 2 = SU/Sahlgrenska, 3 = SU/ Östra |
| Stroke diagnosis | Nominal, Cerebral haemorrhage / infarction | Nominal: 1 = I61, non-traumatic intracerebral haemorrhage, 2 = I 63, cerebral infarction |
| Reperfusion treatment | Binary, yes/no | Binary: 1 = no, 2 = yes |
| NIHSS awareness | Ordinal, 4 levels | Binary: 1 = normal, 2 = impaired |
| NIHSS orientation | Ordinal, 3 levels | Binary: 1 = normal, 2 = impaired |
| NIHSS understanding | Ordinal, 3 levels | Binary: 1 = normal, 2 = impaired |
| NIHSS gaze | Ordinal, 3 levels | Binary: 1 = normal, 2 = impaired |
| NIHSS visual field | Ordinal, 4 levels | Binary: 1 = normal, 2 = impaired |
| NIHSS facial weakness | Ordinal, 3 levels | Binary: 1 = normal, 2 = impaired |
| NIHSS motor arm left | Ordinal, 5 levels | Binary: 1 = normal, 2 = impaired |
| NIHSS motor arm right | Ordinal, 5 levels | Binary: 1 = normal, 2 = impaired |
| NIHSS motor leg left | Ordinal, 5 levels | Binary: 1 = normal, 2 = impaired |
| NIHSS motor leg right | Ordinal, 5 levels | Binary: 1 = normal, 2 = impaired |
| NIHSS ataxia | Ordinal, 3 levels | Binary: 1 = normal, 2 = impaired |
| NIHSS sensory | Ordinal, 3 levels | Binary: 1 = normal, 2 = impaired |
| NIHSS aphasia | Ordinal, 4 levels | Binary: 1 = normal, 2 = impaired |
| NIHSS dysarthria | Ordinal, 3 levels | Binary: 1 = normal, 2 = impaired |
| NIHSS extinction | Ordinal, 3 levels | Binary: 1 = normal, 2 = impaired |
| NIHSS total score | Ordinal, range 0-28p | Ordinal: 1 = NIHSS ≤3 p, 2 = NIHSS 4-8 p,  3 = NIHSS ≥ 9 p |
| Recurrent stroke at stroke units | Binary, no/yes | Binary: 1 = no, 2 = yes |
| Dominant upper limb is affected within 48 h of stroke | Binary, no/yes | Binary: 1 = no, 2 = yes |
| Non - dominant upper limb is affected | Binary, no/yes | Binary: 1 = no, 2 = yes |
| Upper limb problems during the > 48 h post stroke | Binary, no/yes | Binary: 1 = no, 2 = yes |
| Can walk 10 meters independently during the hospital stay | Binary, yes/no | Binary: 1 = Can walk 10 m at discharge, 2 = cannot walk 10 m at discharge |
| Postural balance - sitting | Binary, dependent/independent | Binary: 1 = can sit without assistance, 2 = needs help/supervision during the sitting |
| Postural balance - standing | Binary, dependent/independent | Binary: 1 = can stand without assistance, 2 = needs help/supervision |
| Fall risk at discharge | Binary, no/yes | Binary: 1 = no fall risk, 2 = yes, there is a risk of fall |
| ADL during the hospital stay | Binary, dependent/independent | Binary: 1 = Independent in ADL, 2 = dependent in ADL |
| Mobility during the hospital stay | Binary, yes/no | Binary: 1 = Independent in mobility, 2 = dependent in mobility |
| Level of verbal output | Ordinal, 6 levels | Binary: 1 = normal – patient can be understood, 2 = impaired, the patient needs help during the communication or cannot be understood. |
| Lengths of hospital stay, days | Scale, range 1-100 days | Scale: range 1-100 days |
| Coordinate discharge planning at stroke units | Binary, no/yes | Binary: 1 = no, 2 = yes |
| Discharge destination from the stroke units | Ordinal, 4 levels | Binary: 1 = own home with/without community services, 2 = community facility or other hospitals |
| ADL - activities of daily living, TIA *-* Transient Ischemic Attack, SU - Sahlgrenska university hospital, NIHSS - National Institutes of Health Stroke Scale. | | |

Supplemental Table II. The characteristics of the patients with first-ever stroke included in the Väststroke and Riksstroke registries, stratified based on the reporting stroke unit.

| Features | Admission site, A  (n = 230, 20%) | Admission site, B (n = 513, 46%) | Admission site, C (n = 377, 34%) | P-value |
| --- | --- | --- | --- | --- |
| Sex, n (%) |  |  |  | <0.001 |
| Male | 116 (50) | 320 (62) | 191 (51) |  |
| Female | 114 (50) | 193 (38) | 186 (49) |  |
| Age, years |  |  |  | <0.001^†^ |
| Mean ± s.d. | 76 ± 10.8 | 69 ± 15 | 73 ±11.8 |  |
| Median (range) | 77 (49 -99) | 71 (19-100) | 75 (39-98) |  |
| Stroke type, n (%) |  |  |  | 0.56 |
| I 61 Cerebral haemorrhage | 6 (3) | 17 (3) | 8 (2) |  |
| I 63 Cerebral infarctions | 224 (97) | 496 (97) | 369 (98) |  |
| Stroke severity, n (%) |  |  |  | <0.001^†^ |
| Mild stroke, NIHSS ≤ 3 | 177 (77) | 337 (66 %) | 288 (76 %) |  |
| Moderate stroke, NIHSS 4-8 | 25 (11) | 95 (18%) | 63 (17 %) |  |
| Severe stroke, NIHSS ≥ 9 | 28 (12) | 81 (16 %) | 26 (7 %) |  |
| RLS, fully awake, n (%) | 221 (96) | 491 (96 %) | 368 (98 %) | 0.20 |
| Received reperfusion treatment, n (%) | 3 (1) | 138 (27 %) | 18 (5 %) |  |
| Cognitive screening with the MoCA, n (%) |  |  |  | <0.001 |
| Does not have MoCA | 63 (27) | 257 (50 %) | 168 (45 %) |  |
| Has MoCA  (median, range) | 167 (73) (25, 8-30) | 256 (50 %) (25, 10-30) | 209 (55 %)  (25, 9-30) | 0.52^†^ |
| Length of hospital stay, days |  |  |  | 0.47^†^ |
| Mean ± s.d. | 13 ±12 | 14 ± 16 | 12 ±13 |  |
| Median (range) | 8 (1-86) | 8 (2-93) | 8 (2-100) |  |
| Discharge destination, n (%) |  |  |  | 0.001 |
| Own home with/without community services | 182 (79) | 380 (74 %) | 320 (85 %) |  |
| Community facility /other hospitals | 48 (21) | 133 (26 %) | 57 (15 %) |  |
| Admission site B provides the reperfusion treatment.  Pearson chi-square test and ^†^ Kruskal–Wallis test. P value represent the difference between the groups. MoCA – the Montreal Cognitive Assessment (the scores range from 0-30 points, a low score indicates more severe cognitive deficits), RLS - the Reaction Level Scale NIHSS - the National Institute of Health Stroke Scale (the scores range from 0-42 points, a lower score indicates a less severe neurological status). | | | | |
